# Short-Latency Covert Saccades - The Explanation for Good Dynamic Visual Performance After Unilateral Vestibular Loss?

**DOI:** 10.3389/fneur.2021.695064

**Published:** 2021-08-31

**Authors:** Julia Sjögren, Mikael Karlberg, Craig Hickson, Måns Magnusson, Per-Anders Fransson, Fredrik Tjernström

**Affiliations:** ^1^Department of Clinical Sciences, Otorhinolaryngology Head and Neck Surgery, Skåne University Hospital, Lund University, Lund, Sweden; ^2^Department of Otorhinolaryngology Head and Neck Surgery, William Harvey Hospital, East Kent Hospitals University Foundation Trust, Ashford, United Kingdom

**Keywords:** vestibular loss, compensation, visual performance, covert saccades, saccades, functional head impulse test, video head impulse test, position error

## Abstract

**Background:** Functional head impulse test (fHIT) tests the ability of the vestibulo-ocular reflex (VOR) to allow visual perception during head movements. Our previous study showed that active head movements to the side with a vestibular lesion generated a dynamic visual performance that were as good as during movements to the intact side.

**Objective:** To examine the differences in eye position during the head impulse test when performed with active and passive head movements, in order to better understand the role of the different saccade properties in improving visual performance.

**Method:** We recruited 8 subjects with complete unilateral vestibular loss (4 men and 4 women, mean age 47 years) and tested them with video Head Impulse Test (vHIT) and Functional Head Impulse Test (fHIT) during passive and active movements while looking at a target. We assessed the mean absolute position error of the eye during different time frames of the head movement, the peak latency and the peak velocity of the first saccade, as well as the visual performance during the head movement.

**Results:** Active head impulses to the lesioned side generated dynamic visual performances that were as good as when testing the intact side. Active head impulses resulted in smaller position errors during the visual perception task (*p* = 0.006) compared to passive head-impulses and the position error during the visual perception time frame correlated with shorter latencies of the first saccade (*p* < 0.001).

**Conclusion:** Actively generated head impulses toward the side with a complete vestibular loss resulted in a position error within or close to the margin necessary to obtain visual perception for a brief period of time in patients with chronic unilateral vestibular loss. This seems to be attributed to the appearance of short-latency covert saccades, which position the eyes in a more favorable position during head movements.

## Introduction

The vestibulo-ocular reflex (VOR) stabilizes gaze during head movements, enabling the eyes to maintain focus on visual targets. Hence, if vestibular input is lost, for example after acute unilateral vestibulopathy, other mechanisms will have to be activated in order to maintain visual fixation during head movements. During the acute stage (days) of unilateral vestibular loss, catch-up eye saccades are induced after the head stops moving. Detecting such saccades is the fundament of the clinical head impulse test (1). As these saccades are easily observed by the clinician without supportive equipment, they have been named ‘overt saccades. However, at a later stage of vestibular loss recovery, the catch-up saccades may occur as early as during the head movement. This makes them impossible to perceive without recording equipment and hence they are referred to as ‘covert saccades.

Corrective saccades (overt or covert) are induced to bring the eyes back on target, and are together with a reduced VOR gain (the calculated ratio between eye velocity to head velocity) universally accepted as a sign of VOR hypofunction (2).

During saccades the brain cuts off visual processing, so that neither the motion of the eye (and subsequent motion blur of the image as the world moves across the retina) or the gap in visual perception is perceived by the viewer, phenomena called saccadic suppression and saccadic masking (3).

The mechanism of how covert saccades are induced is still unknown. Previous studies suggest that covert saccades could be triggered by retinal slip (4) or by somatosensory cues from the cervical segment (2). Other activation sources might be residual vestibular function, or other sensory cues that the head is about to, or has just begun to rotate (5) or through generation of internal models by the CNS (i.e., preformed saccades are triggered by expected forthcoming head movements) (6). Recent studies (7, 8) have demonstrated that covert saccades triggered with short latencies reduce oscillopsia and improve visual performance during head movements. VOR function and covert saccades are generally tested with passive head movements, i.e., an examiner rotates the subject's head, so that the tested subject cannot anticipate the beginning or direction of the impulse. When a subject performs active head movements, the covert saccades have even shorter latencies and the subject often exhibit normal visual performance during the head rotation, even when rotating the head toward the side with complete vestibular loss (9).

With video head impulse test (vHIT) systems (10, 11) the examination of the VOR has become more accessible and enabled studies on compensatory mechanisms, such as covert saccades, in detail. Another method for measuring vestibular function is “functional testing”, i.e., to assess how well the VOR performs in respect to its goal of stabilizing gaze and improving visual performance. Usually these tests evaluate the ability to perform a visual task during head rotations, e.g., by displaying different optotypes on a screen. The ability to identify the correct optotype decreases in patients with impaired VOR during head movements due to retinal image slip (i.e., when the image on the retina moves away from the fovea and thus is not in focus). Since visual acuity decreases with increasing angular distance from the point of fixation, i.e. is reduced to 50 % beyond 2° from the fovea (12), minor retinal slippage will reduce visual acuity. Functional tests reflect the combination of the VOR and catch-up saccades on dynamic reading ability (13, 14). Acuity depends also on the distance between the eyes and the visual target, the illumination, and the contrast of the target. However, the ability to perceive the correct optotype is not only influenced by the acuity but by a wide range of parameters such as the shape and color of the stimuli, the attention to the stimuli or the awareness (15) and the duration of the presented stimuli, (with better acuity for longer duration and higher intensities of the presented optotype) (16). The duration of the presented optotype allows for detection at 13 ms (17) and categorization at 68 ms (18). Normal acuities have been demonstrated for high intensity optotypes presented as briefly as 20–30 ms (19, 20).

The aim of this study was to examine the differences in eye position during the head impulse test when performed with active and passive head movements, in order to better understand the role of the different saccade properties in improving dynamic visual performance.

## Methods

### Subjects

Eight subjects (4 men and 4 women) with a mean age of 47 years (range 42–50 years) were recruited for the study: 7 subjects had complete unilateral vestibular loss due to translabyrinthine schwannoma surgery, with the mean time since surgery of 8.6 years (range 1–16 years). One subject had a congenital unilateral deafness and vestibular loss, probably due to an intrauterine cytomegalovirus infection. The complete vestibular loss was confirmed by head impulse tests of all 6 semi-circular canals, bi-thermal caloric tests and cervical vestibular evoked myogenic potentials.

### Ethical Approval

The study was carried out in accordance with Helsinki declaration and approved by the local ethical board (Dnr 2016/32, EPN, Lund University, Sweden). All subjects gave their written and informed consent prior to participation.

### Experimental Protocol

The ability to maintain fixation on a visual reference point while performing rapid head rotations in the horizontal plane, both passively and actively, was assessed with two different methods. (1) The ability to maintain the eye position on a visual reference point during fast accelerations and decelerations of the head was measured using the Interacoustics video head impulse test (vHIT) (EyeSeeCam version 1.2, Interacoustics A/S, Middelfart, Denmark) (21).

(2) The visual performance during rapid head rotations was assessed with the functional head-impulse test (fHIT, Beon Solutions srl, Zero Branco (TV), Italy) (13, 14). The two assessments were performed on the same occasion with a short break between the tests, but the two tests were not performed simultaneously.

### Video Head Impulse Testing

The subject was seated in an arm-less chair 1.5 m in front of a white wall on which a 3 × 3 cm blue marker was placed at eye level, serving as the visual focusing point during the head impulse tests. The subject was instructed to keep visual fixation on the blue marker at all times during the assessment. All head impulse tests were carried out by the same examiner who stood behind the subject, imposing manual, fast head rotations in the horizontal plane with peak velocities exceeding 150°/s, accelerations/decelerations chiefly within 3,000–8,000°/s^2^ and a movement amplitude of about 10–25°. The vHIT testing continued until the software had accepted at least 10 passive head-impulses in each direction according to the criteria above. After testing the VOR with passive head movements, the subject was instructed to perform active, self-generated head rotations with similar velocity, acceleration and amplitude as were used during passive head rotations. The subjects were allowed to practice their active head movements until they felt comfortable, which normally included only a few trials before such an everyday-like movement was easy to execute.

The vHIT testing proceeded in the same manner as before, until the software had accepted 10 impulses in each direction (i.e., the head movement performance criteria imposed by the software were identical during active and passive head movements). The EyeSeeCam system records eye and head movements at a sample frequency of 220 Hz, whilst the analyses of the recorded data were based on signal-processed data elevated to the sampling frequency of 1.000 Hz by the software (21).

### Functional Head Impulse Testing

Functional tests of visual performance during ongoing head movement was performed with the fHIT device (Beon Solutions srl, Zero Branco (TV), Italy). The subject was seated 1.5 m in front of the fHIT computer screen, wearing a head mounted accelerometer and holding a keyboard in their hands with Landolt C optotypes in 8 different orientations. The f-HIT monitor was full HD with a refresh rate of 60 Hz and set to the 1920 × 1080 resolution. First a setup session was performed by an assessment of static visual acuity, where the subject viewed scaled optotypes. The subject was required to choose the correct orientation of a sequence of Landolt C optotypes in one of the eight possible positions by pressing the corresponding button on the keyboard after a brief display of the symbol. The acuity assessment started from a symbol size of 1logMAR (log of the Minimum Angle of Resolution). Thereafter the size of the optotypes gradually decreased depending on the rate of errors made by the subject over 20 trials. The subsequent fHIT tests were performed using an optotype size calculated from the size of the smallest correctly detected optotype multiplied by a factor of 0,8logMAR.

In the first fHIT test session, the examiner manually imposed head impulses until the fHIT software had accepted 10 impulses in each direction. The fHIT software criteria for a valid head impulse (active and passive) was an acceleration ranging from 3,000–6,000 °/s^2^.

The subject was instructed to focus on a dot presented on the fHIT screen. When the head thrust was performed, the software continuously scanned the recorded data for when the angular velocity of the head exceeded 10°/s and an acceleration 300°/s^2^. Passing these thresholds defined the start of a head thrust and made the software present, with a delay of about 62 ms, a random Landolt C in one of eight possible orientations to the subject, during a 83 ms period on the fHIT-screen (14). The subject was requested immediately after the completed head thrust to choose the symbol they perceived by pressing the corresponding button on the keyboard. In the second fHIT session, the same tests were performed but the head thrusts were actively made by the subjects themselves in the same manner as for the vHIT tests. The subjects were allowed to practice their active head movements until they felt comfortable, which normally included only a few trials before such an everyday-like movement was easy to execute.

### Data Analysis

The eye positioning performance during the vHIT tests was assessed by a customized software. The Interacoustics vHIT hardware records the movements of the eyes and head using two different methods. The eye movements are recorded by a video system and the head movements by an accelerometer device. Thus, as a first step, it was ensured that for each trace the recorded movements of the head and eyes matched in amplitude. The analysis was performed by calculating the position data of the eyes and head during a 700 ms analysis window, using an integration formula on the velocity data recorded by the two devices. The time frame of this analysis window included about 50 ms of data prior to the start of head movement (see **Figures 2C,D**, displaying the first 400 ms of the 700 ms data). The total head and eye movements made, as recorded individually by each device, was thereafter determined by defining stable starting and finishing positions of the eyes and head, and by calculating the distance moved by subtracting the final position from the starting position. Finally, using the head position data as reference, an amplitude adjustment was made to the eye position data on sample levels, so that the resultant trajectory produced an identical total movement of the eyes and head. These normalized eye and head position data were thereafter used in the subsequent stages of the data analysis, e.g., when needed, the velocity and acceleration data was produced by deriving the normalized eye and head position data.

The absolute mean error between the normalized eye position and recorded head position was calculated and analyzed during three time frames:

1. The total head movement, where the start was defined as when the head velocity exceeded 10°/s and the end when the head velocity decreased below 10°/s.
2. The delay, where the start was defined as when the head velocity exceeded 10°/s and the end as 62 ms later (matching the criteria used in the fHIT test), see **Figure 2**.
3. The optotype presentation, with start 62 ms after when the head velocity exceeded 10°/s and end defined as 80 ms later (matching the criteria used in the fHIT test). In the fHIT test it is during this time frame that the optotype is presented to the subject.

The software also assessed whether the normalized eye velocity data included saccades, and if so, the time from when the head movement started until the first saccade reached peak velocity (peak latency), and what velocity the saccade had at its maximum (peak velocity). The start of the head movement was defined as when the head movement velocity exceeded 30°/s. An eye movement was defined as a saccade if the following criteria were fulfilled: 1) The peak velocity exceeded 80°/s; 2) Both the acceleration and deceleration phases exceeded 3,000°/s^2^ and 3) The saccade duration was within the range of 10–80 ms.

The fHIT data was presented as the percentage of correctly identified optotypes as calculated by the fHIT 1.0 software system.

The equipment used did not allow vHIT and fHIT tests to be performed simultaneously. An off-line synchronization approach was instead applied. Hence, a series of fHIT and vHIT tests were carried out independently and the performance during the vHIT were analyzed using the inclusion and analysis criteria applied during the fHIT tests, in order to obtain matching eye position error data from the vHIT recordings. During the fHIT test, the software presented, with a delay of about 62 ms ±17ms, a random Landolt C in one of eight possible orientations to the subject during a 83 ms period on the fHIT-screen (14). Thus, with a random uncertainty of a maximum of ±17 ms (i.e., the optotype can be presented both before and after 62 ms) from the random frame rate update, during the denoted “optotype presentation” time period in the vHIT-analysis, the optotype would have correspondingly been visible in the fHIT test during at least 80% of this time.

### Statistical Analysis

Saccade peak latency, saccade peak velocity and the absolute position errors of the eyes during the three different time frames; total head movement, the delay, and the optotype presentation, were analyzed using repeated measures GLM ANOVA (General Linear Model Analysis of Variance) (22). The main factors and factor interactions analyzed were: active vs. passive head movements; d.f. 1, and ipsilesional vs. contralesional head movement direction; d.f.1. The repeated measures GLM ANOVA analysis was used after ensuring that all model residuals had normal or approximate normal distribution (22). Hence, the fHIT datasets did not fulfill the criteria to be analyzed by GLM ANOVA.

The Wilcoxon matched-pairs signed-rank test (Exact sig. 2- tailed) was used for within-group comparisons, i.e., analyzing the difference between ipsilesional and contralesional head movement responses during active and passive head movements (22). These analyses were performed for all parameters; fHIT-score, saccade peak latency, saccade peak velocity and the eye position errors during the total head movement-, the delay- and the optotype presentation-time frame.

Spearman correlations were performed to determine any relationships between parameters during head movements in the ipsilesional and contralesional directions. These correlation datasets included values from active and passive head rotations merged. The correlations were calculated for the parameter combinations; fHIT-score, saccade peak latency, saccade peak velocity and the eye position error during the total head movement-, the delay- and the optotype presentation-time frame.

For the GLM ANOVA and Spearman correlation analyses, *p*-values < 0.05 were considered significant. In the Wilcoxon analyses, *p*-values < 0.025 were considered significant after Bonferroni correction. The non-parametric Wilcoxon test and Spearman correlation tests were used since the Shapiro-Wilk test revealed that some datasets were not normally distributed and normal distribution could not be obtained by log-transformation.

## Results

Passive head movements toward the lesioned side generated covert saccades in 79 ± 11% (mean, SEM) of the impulses, whereas active movements generated covert saccades in 98 ± 1% (mean, SEM) of the impulses. For contralesional movements the percentage of generated covert saccades was 31 ± 13% (mean, SEM) for passive movements and 37 ± 13% (mean, SEM) for active movements.

**Figure 1:**
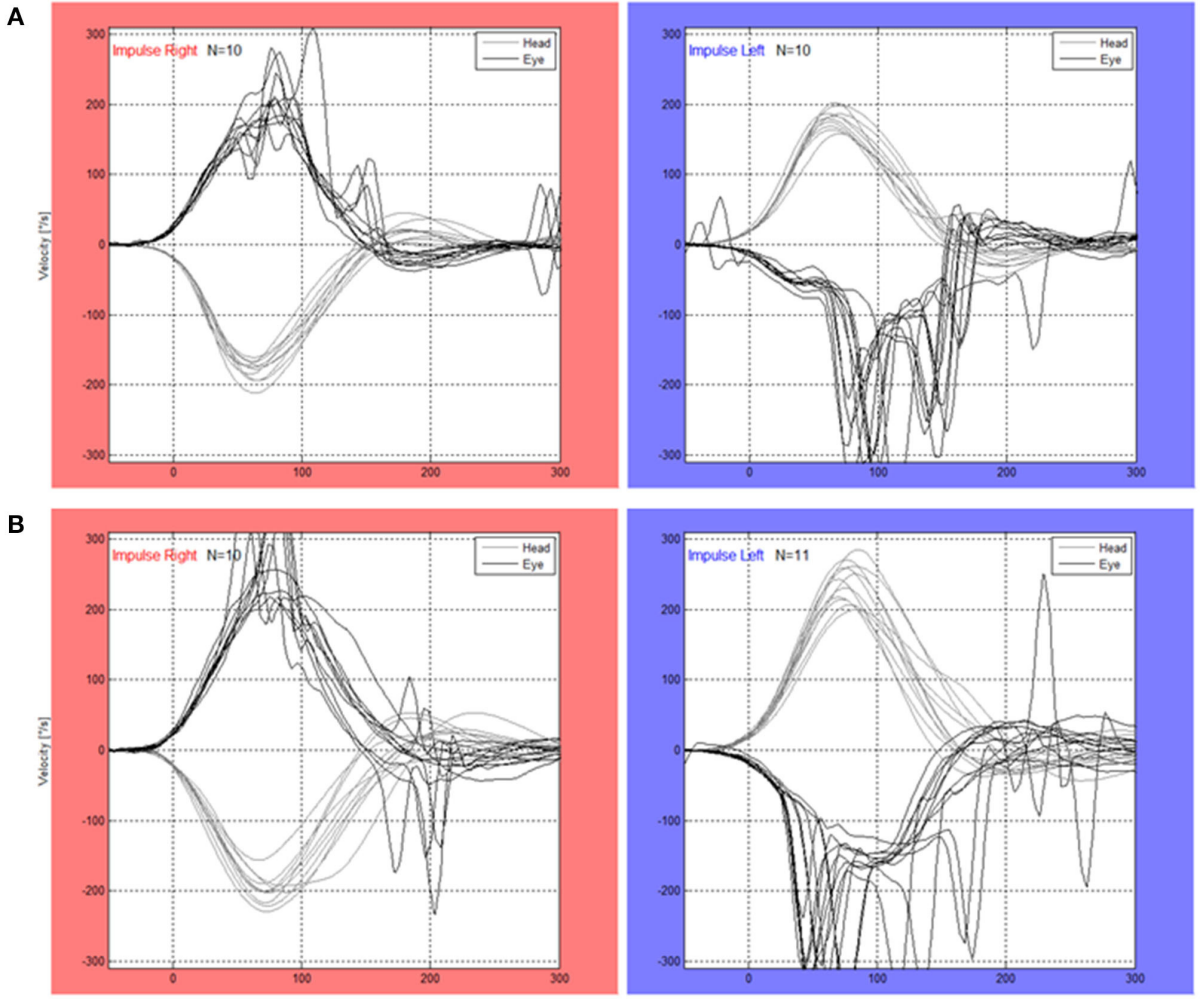
A raw data image from the Interacustics software from one representative subject during passive head impulses **(A)** and active head impulses **(B)** toward the right side (contralesional movements) and left side (ipsilesional movements).

**Figure 2:**
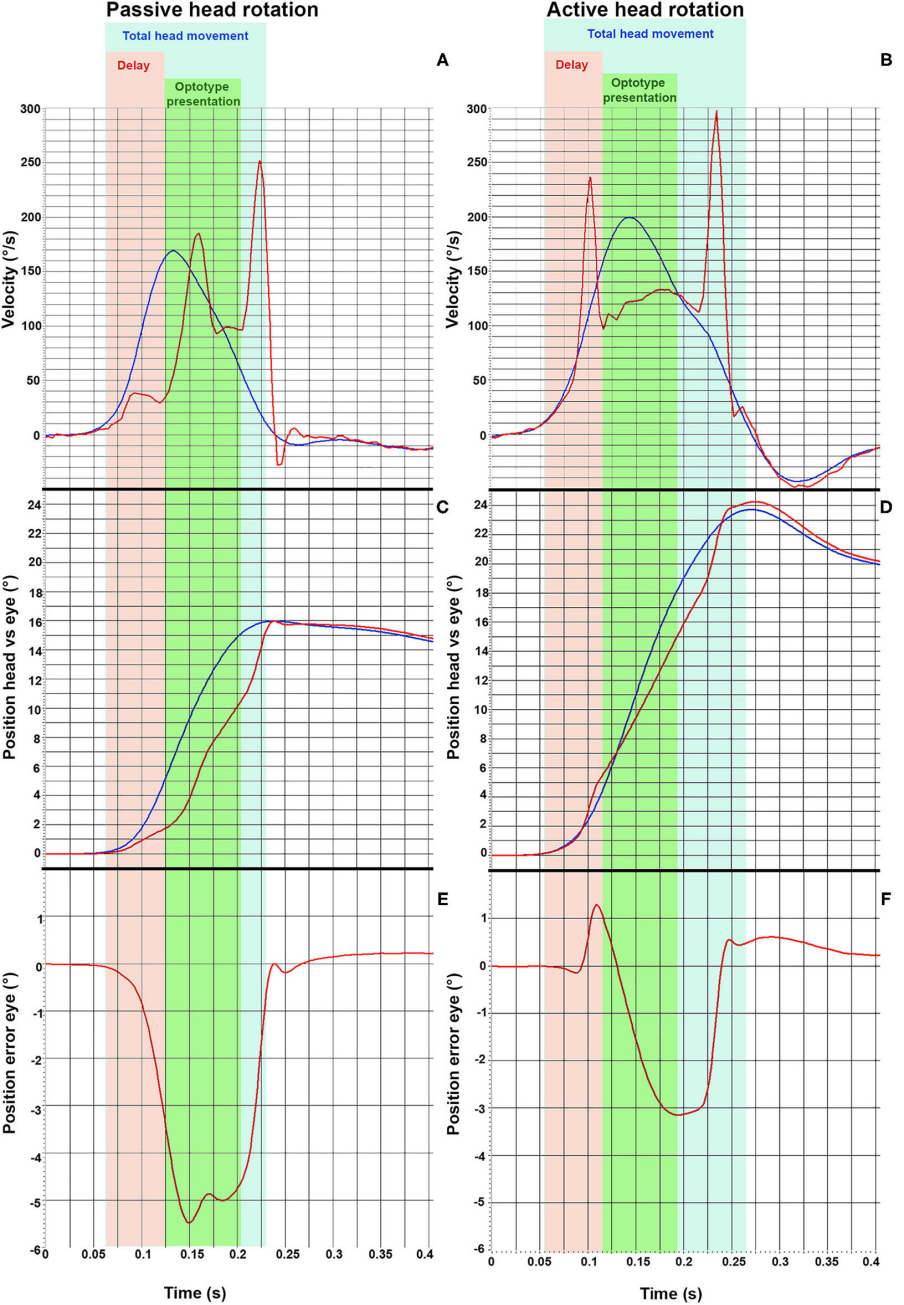
vHIT traces from one representative subject during a passive head impulse **(A,C,E)** and during an active head impulse **(B,D,F)** toward the lesioned side (ipsilesional). The head velocity (blue line) and the eye velocity (red line) during the head thrust are displayed in **(A,B)**. The angular position of the head and of the eyes are displayed in **(C,D)**. Finally, the error between the angular position of eyes with respect to the head position are displayed in **(E,F)**, where a negative value indicates that the eye movement lags behind the head movement. The different time frames analyzed are presented as different colors. The light blue area represents the total head movement. The orange field demarks the delay (62 ms) and the green area demarks the optotype presentation, during which the optotype to be identified is presented to the subject (80 ms).

Passive (A) and active (B) vHIT-traces from one representative subject are shown in Figure 1. Figure 2 displays a single passive (A, C, E) impulse and a single active (B, D, F) impulse toward the lesioned side from the same representative subject as in Figure 1.

Both passive and active head-impulses toward the lesioned side (ipsilesional head movements) induced two covert saccades, but with noticeably shorter latencies during the active head movement (Figures 2A,B). Both kinds of head thrust produced a position error between the head and eye, representing that the eyes drifted away from the visual target (Figures 2C,D). However, the saccades during the active head movement were performed so early that the position error of the eyes was small during the time frame when the optotype was displayed (Figures 2E,F), and thus, enabled the subjects to determine the orientation of the optotype.

### GLM-ANOVA and *post-hoc* Wilcoxon Analysis on Eye Position Performance

**Figure 3:**
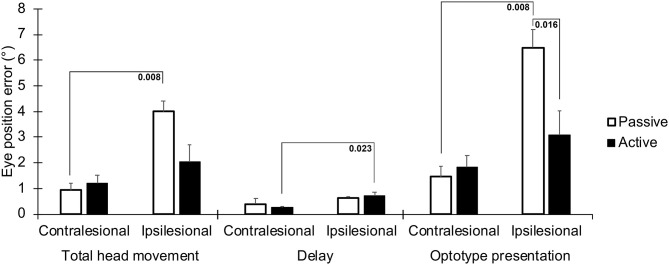
Absolute position errors between the eyes and head, representing a drift of the eyes from the visual target the subjects were instructed to focus on during the head movement. Values are presented for the three time frames; during the total head movement, during the delay, and during the optotype presentation, for head movements in the ipsilesional and contralesional directions during active and passive head movements. Bars representing mean values for the group. Error bars indicate SEM. Bonferroni corrected post-*hoc* statistics presented numerically.

The position error of the eyes was significantly greater for movements toward the lesioned side in all three time frames, especially during passive impulses (Figure 3 and Table 1). During the time when the optotype was presented, the difference in position error between active and passive head movements was the greatest (*p* = 0.016, Figure 3). The interaction analysis in Table 1 further supports that the difference in position error was the largest during passive movements toward the lesioned side during optotype presentation (*p* = 0.006).

**Table 1:** Repeated measures GLM ANOVA of anteroposterior spectral power with main factors “active vs. passive” and “contralesional vs. ipsilesional” and their factor interactions.

Parameters^*^		**Passive/Active**	**Contra/Ipsilesional**	**Passive/Active x Contra/Ipsilesional**
Position error	Total head movement	0.058 [5.2]	**0.003 [20.9]**	**0.016 [9.9]**
	Delay	0.745 [0.1]	**0.006 [14.8]**	0.523 [0.5]
	Optotype presentation	**0.033 [7.1]**	**0.001 [25.7]**	**0.006 [15.3]**
Saccade properties	Peak latency	0.119 [3.2]	**0.023 [8.5]**	0.408 [0.8]
	Peak velocity	0.060 [5.0]	**0.004 [17.6]**	0.226 [1.8]

* *F-values are presented in the squared parenthesis*.

*Bold statistics denote p-values < 0.05*.

During the total head movement, the eye position error was significantly larger during passive head movements toward the ipsilesional side when compared with head movement toward the contralesional side (*p* = 0.008, Figure 3). During the delay, the eye position error was significantly larger during active head movements toward the ipsilesional side when compared with head movements toward the contralesional side (*p* = 0.023, Figure 3).

When the optotype was presented, the eye position error was significantly larger during passive head movements toward the ipsilesional side when compared with head movements toward the contralesional side, (*p* = 0.008, Figure 3). Moreover, the eye position error was significantly (*p* = 0.016) larger during head movements toward the ipsilesional side with passive head movements when compared with actives head movements.

**Figure 4:**
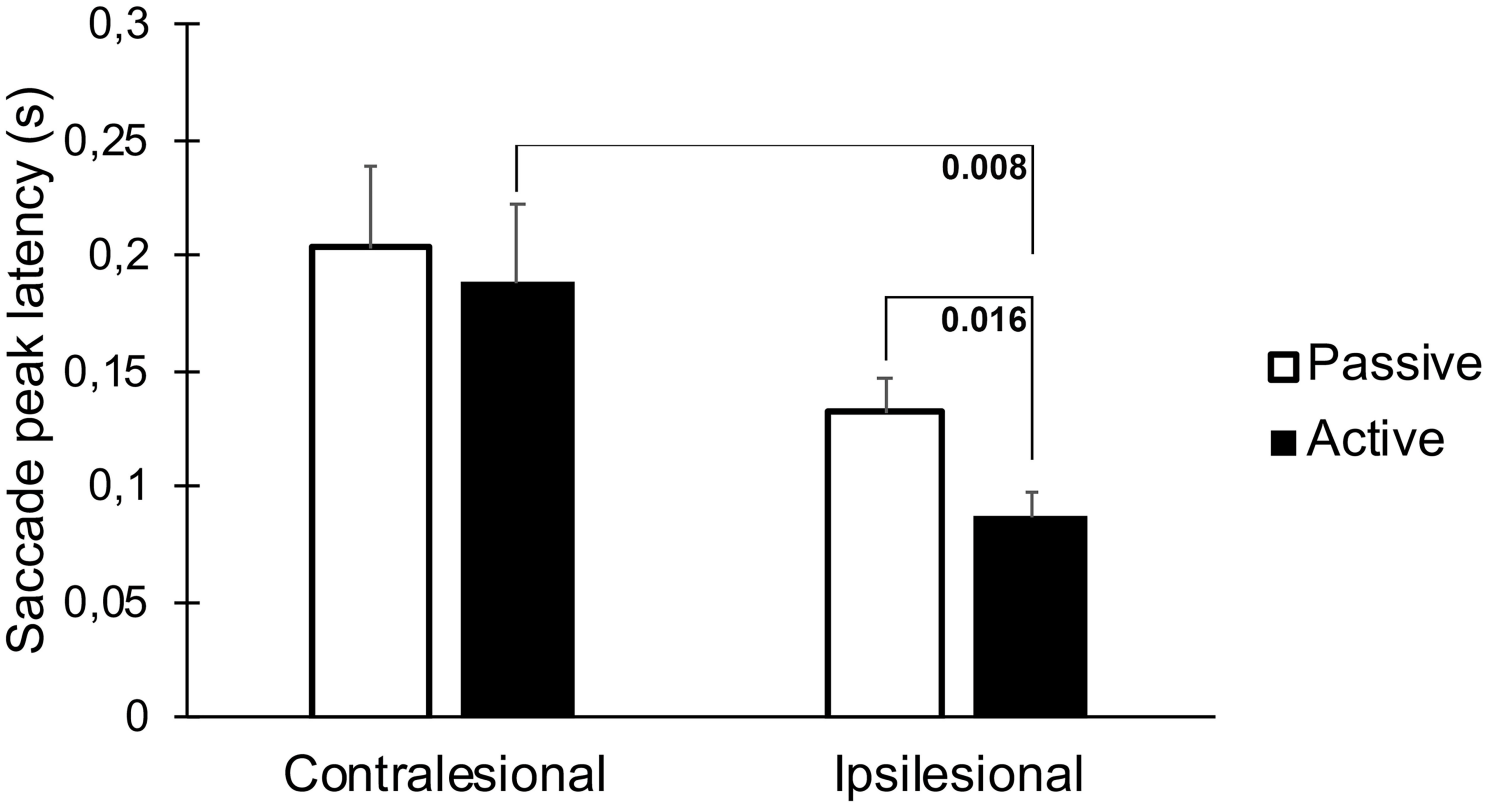
Latency to the first saccade when performing head thrusts in the ipsilesional and contralesional directions with active and passive head movements. Bars representing mean values for the group. Error bars indicate SEM. Bonferroni corrected post-*hoc* statistics presented numerically.

**Figure 5:**
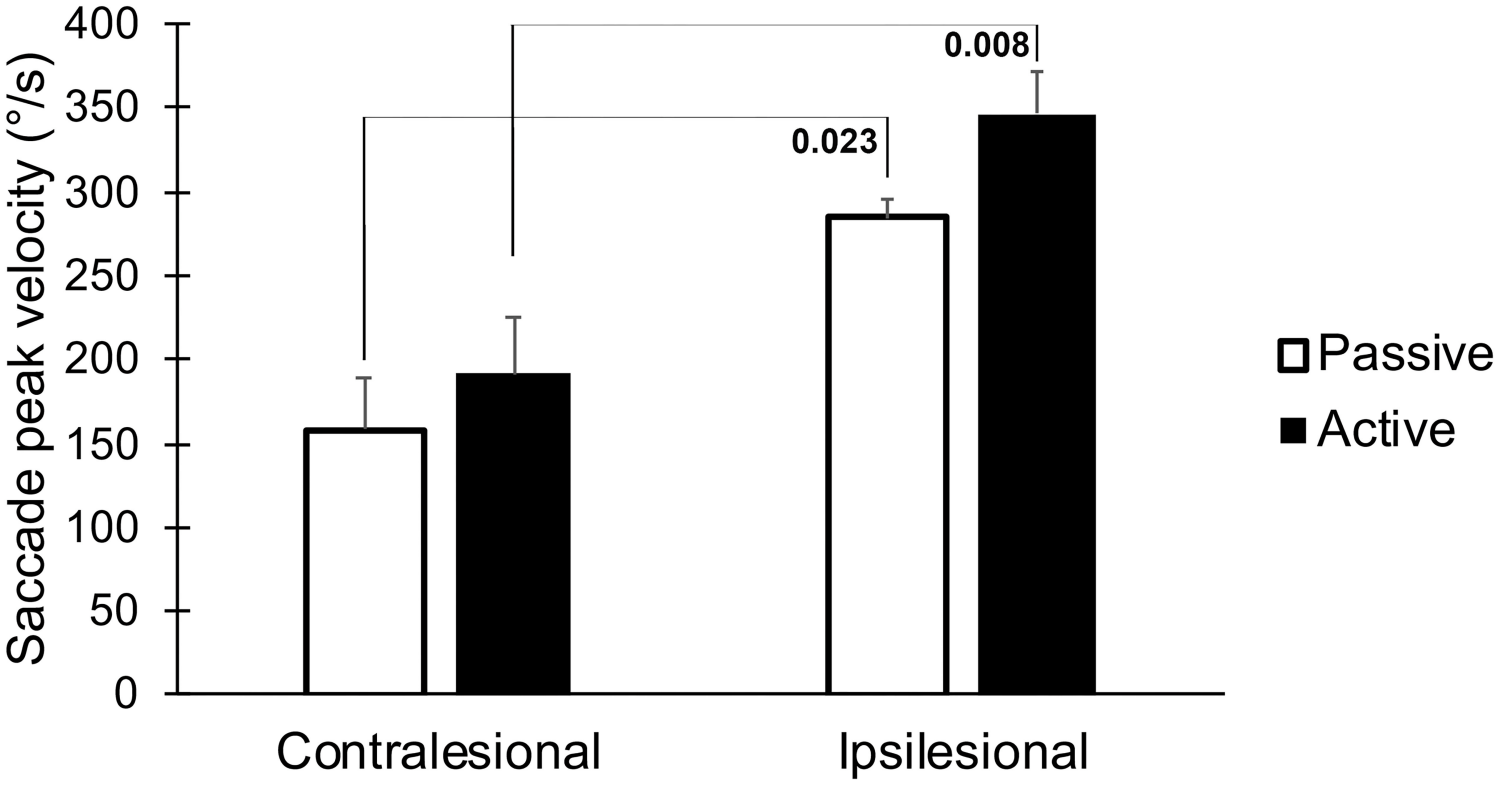
The peak velocity of the first saccade, when performing head thrusts in the ipsilesional and contralesional directions with active and passive head movements. Bars representing mean values for the group. Error bars indicate SEM. Bonferroni corrected post-*hoc* statistics presented numerically.

The latencies to the first saccades were significantly shorter if the movement occurred toward the lesioned side (*p* = 0.023, Table 1, Figure 4). These movements also generated greater saccade velocities (*p* = 0.004, Table 1, Figure 5). The latency was further decreased if the movement was actively induced when compared to passive movement (*p* = 0.016, Figure 4).

**Figure 6:**
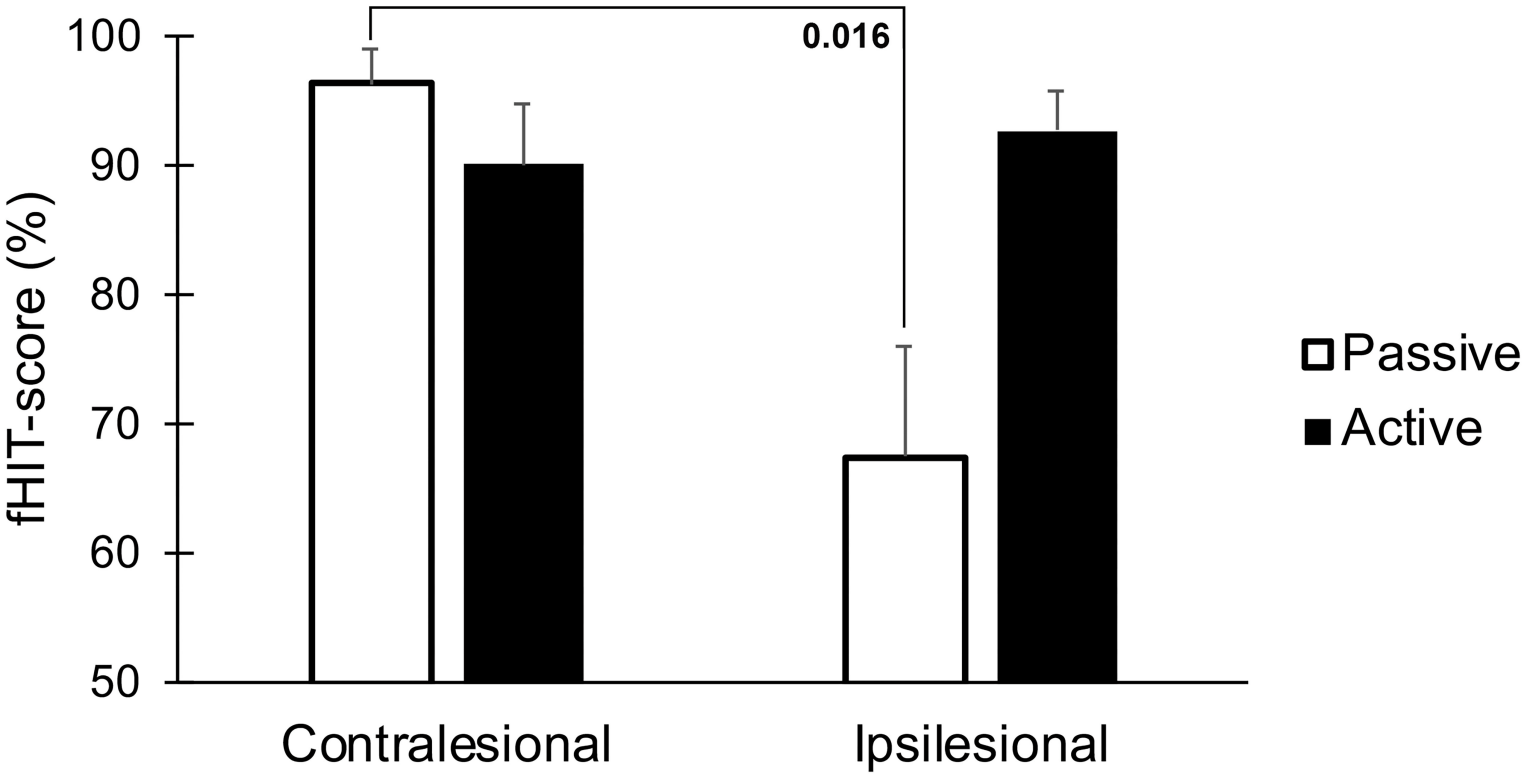
Accuracy of visual perception as assessed by the fHIT test when performing head thrusts in the ipsilesional and contralesional directions with active and passive head movements. Bars representing mean values for the group. Error bars indicate SEM. Bonferroni corrected post-*hoc* statistics presented numerically.

The fHIT-score was significantly lower during passive head movements toward the lesioned side when compared to the healthy side, but notably not different during active movements (*p* = 0.016, Figure 6).

### Between-Parameter Relationships During Head Movements to the Ipsilesional and Contralesional Side

A strong correlation was found between saccade latencies and position errors during total head movements (*p* < 0.001; Table 2) and during the optotype presentation time frame for ipsilesional movements (*p* < 0.001). These correlations were not found for contralesional movements. The fHIT-score negatively correlated to the position error during the optotype presentation phase, with lower fHIT scores when the position error was large (*p* = 0.023, Table 2). The correlation analysis further revealed that when the head thrusts were performed toward the contralesional side, the saccades that appeared had significantly shorter latency times if the saccade peak velocities were large (*p* = 0.003, Table 2).

**Table 2:** Spearman correlation analyses of between-parameter relationships during head movements in the ipsilesional and contralesional directions, active and passive movements merged.

Parameters^*^		**Ipsilesional**			**Contralesional**	
		**Saccade peak latency**	**Saccade peak velocity**	**fHIT- score**	**Saccade peak latency**	**Saccade peak velocity**	**fHIT- score**
Position error	Total head movement	** <0.001[0.808]**	0.322 [−0.265]	0.065[−0.472]	0.345 [−0.253]	0.154[0.374]	0.182 [−0.352]
	Delay	0.324[0.263]	0.284 [0.285]	0.969[0.011]	0.807 [−0.066]	0.305[0.274]	0.719 [−0.098]
	Optotype presentation	** <0.001[0.915]**	0.770 [−0.079]	**0.023[**–**0.565]**	0.264 [−0.297]	0.119[0.406]	0.140 [−0.385]
Saccade properties	Peak latency	–	0.961 [−0.013]	0.054[−0.491]	–	**0.003[**–**0.67]**	0.717 [0.098]
	Peak velocity	0.961[−0.013]	–	0.698[−0.105]	**0.003 [**–**0.697]**	–	0.817 [0.063]
fHIT-score		0.054[−0.491]	0.698 [−0.105]	–	0.717 [0.098]	0.817[0.063]	–

* *The correlation coefficients are presented in the squared parenthesis*.

*Bold statistics denote p-values < 0.05*.

## Discussion

Passive head rotations toward the side with the complete vestibular loss resulted in substantial eye position errors, while actively generated head rotations yielded significantly smaller errors. As stated in the introduction the ability to properly detect optotypes declines rapidly for each degree the image falls outside the fovea (12) and recognition of images can be as fast as 13 ms (17). The main reason for the modest position error generated during active head rotations during the optotype perception time frame seems to be the shorter latency of the first covert saccade. This finding might explain the results from our previous study (9), where subjects with long-standing uVL had almost normal dynamic visual performance during actively generated head movements, even for rotations toward the side with a complete vestibular loss. This is consistent with previous findings that saccades are triggered earlier during self-generated head turns (23, 24), and that the saccade latency correlates with dynamic visual performance (7, 25). Yet, since there is supposed to be no visual input during saccadic eye movements due to saccadic suppression (3), the reason for improved visual performance is not only that saccades are generated, but that they have to be elicited early enough to replace a deficient VOR. The covert saccades coupled with self-generated movements had latencies so short that feedback reflexes were probably to slow to have triggered them, rather they could be interpreted as having cortical origin (feed-forward mechanism).

This is, to our knowledge, the first study seeking to determine the positional status of the eyes coupled with covert saccades during active and passive head rotations.

One of the chief complaints for patients with vestibular loss is blurred vision during quick head rotations, when walking or when running. Hence, improving gaze stability is one of the central objectives of vestibular rehabilitation. Gaze stability exercises have been proven to increase VOR gain for active head movements (26–28), and it has been suggested that the increase in VOR gain explains the subsequent improved dynamic visual performance (29). However, the majority of those without gain improvement after gaze stability exercises also report less symptoms while walking (30). This suggests that improved VOR gain may not be the only reason for functional improvement. Furthermore, it has been reported that the presence of predominately covert saccades (31) and the organization of covert saccades in patients with longstanding uVL is more important for dynamic visual performance. Highly organized corrective saccades, i.e., that occur in a constant fashion impulse after impulse, correlate to lower Dizziness Handicap Inventory (DHI) scores (32). A high ratio of covert saccades also correlates with better performance on measures of dynamic visual performance, gait, and balance (31).

Our material was too small to evaluate subjective parameters such as DHI score but we found strong relationships between active head movements, short latency covert saccades and moderate position errors during the optotype presentation, which might explain an almost normal dynamic visual performance despite a complete vestibular loss (9). The present findings propose an explanation for this phenomenon. The development of covert saccades appears to be a crucial part of vestibular rehabilitation and there is increasing evidence for their association with a better compensation. Hence, vestibular rehabilitation should also be designed to drive the development of short-latency covert saccades.

The mechanism of how covert saccades are generated is still under debate. Corrective saccades triggered by remaining vestibular function or by somatosensory cues from the cervical segment has been suggested (2). Retinal slip during the onset of head movement as the main trigger of corrective saccades is another possible explanation (4, 33). Yet, the presence of corrective saccades in darkness (33) and covert saccades with almost no latency at all (9), indicates that there must be other triggers than retinal slip. Another explanation might be that the CNS in well compensated patients has learned to instigate preformed saccades coupled with head movements of certain amplitudes in anticipation. This is consistent with previous findings that covert saccades are triggered earlier during self-generated head turns (23, 24), and as displayed in Figure 2F, that the eyes are positioned pre-emptively in a way to address the forthcoming drift in position during the later phase of the head thrust.

One might argue that the head and eye movement during the active head impulse test is not very “natural” from a biological point of view. Rather than focusing your eyes in the center, you would want to focus your eyes on to an oncoming object at the periphery and keep them locked on target while rotating the head toward the side. For gaze shift movements, the eyes start to move 25–40 ms prior to the head if an unpredictable target is flashed in the periphery (34). The opposite is true for predictable targets, the head will start to move several hundred milliseconds before the eyes and both the eye and head will commence to move even before the visual target moves itself (35). In our set-up, the subject knew the location and timing of the flashed optotype, i.e., where to focus the eyes during the head movement. During all the years of practice, patients with longstanding uVL might have developed an anticipatory mechanism, opposite to that of the normal gaze shift paradigm, that command the eyes to move prior to the head, and thus, position the eyes in a more favorable position enabling foveation despite a defective VOR. In everyday life targets are of an unpredictable nature, why the paradigm of gaze shift with an unpredictable target; i.e. moving the eyes prior to the head, would be more “natural” and this might be what happens when the optotype is flashed. Thus, the CNS triggers a preformed gaze shift with the eyes moving much faster than the head. This behavior would be advantageous, for example when turning a corner, in that the head and eyes are directed toward the oncoming visual scene and this anticipatory mechanism may reflect the need to prepare a stable reference frame for an intended action (36).

If the actively generated saccades are anticipatory and of cortical origin, why do some impulses yield more than one saccade and why do the saccades have any latencies at all? For patients with longstanding uVL we propose that the first covert saccade might be anticipatory and preformed in order to place the eyes in a more favorable position during the early phase of the head movement. We also propose that the second and other subsequent saccades are adaptive, triggered and controlled in size by the retinal slip to refocus the eyes perfectly on the visual target. To understand the mechanisms behind covert saccades, more prospective research is needed that in larger cohorts assess the course of active and passive fHIT and vHIT performance over time after uVL, preferably together with subjective estimates of perceived vestibular function, e.g., by using questionnaires like the DHI and Vertigo Symptom Scale.

### Limitations

One weakness of the present study is the relatively small sample size, which makes it difficult to analyze subjective parameters. Moreover, the relatively small age-span in the material investigated makes it difficult to generalize the results to populations of other ages. Nonetheless, we could determine a strong relationship between self-generated head rotations, short latency covert saccades and position errors within the time frame to obtain visual perception during the optotype presentation phase, which is the probable explanation for the better fHIT results during self-generated movements (9).

Our material comprised only well-compensated subjects, of whom all but one (congenital uVL) had performed vestibular rehabilitation after vestibular schwannoma surgery. This is of importance when comparing compensational strategies, since these evolve over time. Further studies, investigating the development of compensational strategies from an acute onset of a vestibular loss to full compensation, may establish how saccadic strategies develop.

In this study, the eye tracking vHIT tests and the functional tests with fHIT were not recorded during the same head thrusts. This makes comparisons between data from the vHIT testing, such as position error, and data from the fHIT tests such as perception performance, problematic. On the other hand, the analyses criteria were identical for the fHIT and vHIT tests, and thus, the assessment setup and mathematical criteria defining the different phases analyzed during a head thrust were identical.

### Conclusions

Actively generated head impulses toward the side with a complete vestibular loss resulted in a position error within or close to the margin necessary to obtain visual perception for a brief period of time in patients with chronic unilateral vestibular loss. This seems to be attributed to the appearance of short-latency covert saccades, which position the eyes in a more favorable position during head movements.

## Data Availability Statement

The raw data supporting the conclusions of this article will be made available by the authors, without undue reservation.

## Ethics Statement

The studies involving human participants were reviewed and approved by Regionala Etikprövningsnämnden Lund (EPN). The patients/participants provided their written informed consent to participate in this study.

## Author Contributions

FT and P-AF contributed to conception and design of the study. JS carried out the data collection. JS and P-AF performed the statistical analysis. JS, FT, P-AF, MK, and MM interpreted the results. JS wrote the first draft of the manuscript. All authors contributed to manuscript revision, read, and approved the submitted version.

## Conflict of Interest

The authors declare that the research was conducted in the absence of any commercial or financial relationships that could be construed as a potential conflict of interest.

## Publisher's Note

All claims expressed in this article are solely those of the authors and do not necessarily represent those of their affiliated organizations, or those of the publisher, the editors and the reviewers. Any product that may be evaluated in this article, or claim that may be made by its manufacturer, is not guaranteed or endorsed by the publisher.
